# Leber’s hereditary optic neuropathy, intellectual disability and epilepsy presenting with variable penetrance associated to the m.3460G >A mutation and a heteroplasmic expansion of the microsatellite in *MTRNR1* gene – case report

**DOI:** 10.1186/s12881-018-0644-3

**Published:** 2018-07-27

**Authors:** Angelica Bianco, Luigi Bisceglia, Maria Fara De Caro, Valeria Galeandro, Patrizia De Bonis, Apollonia Tullo, Stefano Zoccolella, Silvana Guerriero, Vittoria Petruzzella

**Affiliations:** 1 Dipartimento di Scienze Mediche di Base, Neuroscienze e Organi di Senso, Università degli Studi Aldo Moro, Piazza G. Cesare, 70124 Bari, Italy; 2 Ospedale Casa Sollievo della Sofferenza IRCCS, UOC Genetica Medica, San Giovanni Rotondo, Italy; 3 Istituto di Biomembrane, Bioenergetica e Biotecnologie Molecolari, IBIOM – CNR – Via G, Amendola 165/A, 70126 Bari, Italy

**Keywords:** LHON, Penetrance, Intellectual disability, Mitochondrial DNA, MTRNR, M.3460G > A

## Abstract

**Background:**

Leber’s hereditary optic neuropathy (LHON) associated with mutations in mitochondrial DNA (mtDNA) typically manifests only optic nerve involvement but in some patients may develop additional neurological complications. The cause of this association is not clear.

**Case presentation:**

We present a case of a 24-year-old male with a history of subacute, painless, and rapidly progressive bilateral vision loss. We performed ophthalmological, neurological and neuropsychological investigations in the proband and his LHON family. The proband showed optic neuropathy, epilepsy, migraine, and intellectual disability; all the maternal relatives did not manifest optic neuropathy but a moderate to severe intellectual disability. Genetic screening revealed a novel association of the LHON m.3460G > A primary mutation with the m.T961delT + C(n)ins within the mitochondrial encoded 12S RNA (*MTRNR1*) gene which segregates with the intellectual disability through the maternal branch of the family. We also found a significant increase of mtDNA content in all the unaffected homo/heteroplasmic mutation carriers with respect to either affected or control subjects.

**Conclusion:**

This is the first case reporting the co-segregation of a mutation in *MTRNR1* gene with a LHON primary mutation, which may be a risk factor of the extraocular signs complicating LHON phenotype. In addition, the data herein reported, confirmed that the key factor modulating the penetrance of optic atrophy in the family is the amount of mtDNA.

**Electronic supplementary material:**

The online version of this article (10.1186/s12881-018-0644-3) contains supplementary material, which is available to authorized users.

## Background

Leber’s Hereditary Optic Neuropathy (LHON), is due to three primary mutations (i.e. m.3460G > A in *MT-ND1*, m.11778G > A in *MT-ND4* and m.14484 T > C *MT-ND6*) in the mitochondrial DNA (mtDNA) genes, encoding for three different subunits of NADH: ubiquinone oxidoreductase (EC 1.6.5.3) or complex I (CI) for more than 90% of cases [1]. LHON is typically characterized by a rapid bilateral central vision loss owing to focal degeneration of the retinal ganglion cell layer and optic nerve [2, 3]. Typically, not all the individuals who inherit LHON primary mutations develop optic neuropathy and visual impairment [4] thus accounting for the extremely variability of penetrance in families. In some cases, the ophthalmological signs may be complicated by additional neurological signs such as dystonia [5–8], parkinsonism [5], cerebellar ataxia [9, 10], epilepsy [11, 12], myoclonus [13, 14], juvenile-onset encephalopathy and psychiatric disturbances [1]. Neither the highly variable penetrance of the optic neuropathy ─ which is always much greater in males ─ or the presence of extraocular signs can be exclusively explained by the presence of primary mutations that are necessary but not sufficient to cause the disease. All these elements suggest that other genetic and/or environmental factors must influence the phenotype. From a genetic standpoint, the mtDNA itself may contribute in various ways to LHON manifestation: the homo- or heteroplasmy conditions of the primary mutation [15, 16]; the presence of additional mtDNA mutations [17]; the number of mtDNA copies within the cells [18–21].

Herein, we assessed the presence and the homo/heteroplasmic *status* of the m.3460G > A LHON mutation; the co-occurrence with novel mutation m.T961delT + insC(n) within the mitochondrial encoded 12S RNA (*MTRNR1*) gene and the mtDNA cellular content in a family manifesting ocular neuropathy complicated by intellectual disability, migraine and epilepsy as non-ophthalmologic features.

## Case presentation

**Fig. 1:**
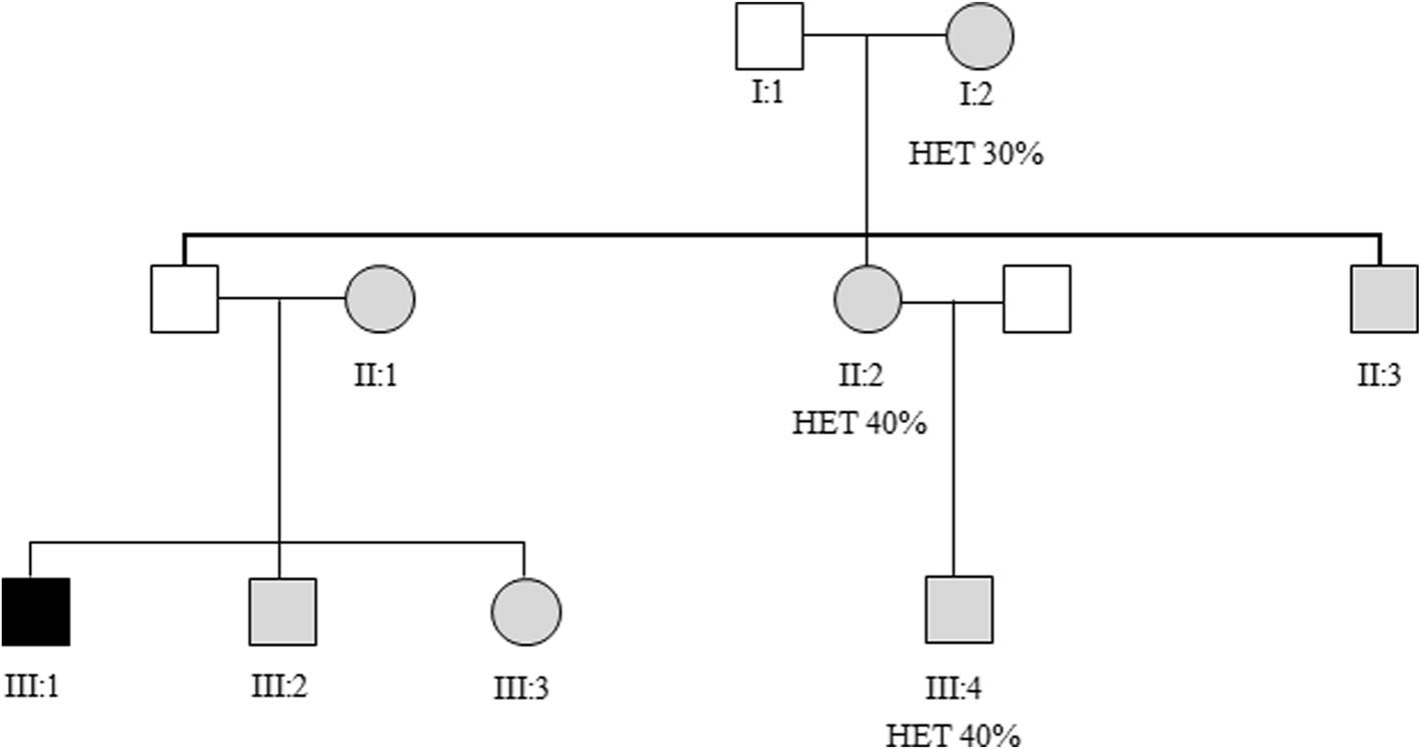
Pedigree of the m.3460G > A family. The black symbol indicates the proband; gray symbols indicate asymptomatic relatives. Percentages of heteroplasmy of m.3460G > A mutation are reported

The proband, a 24 year old male from Southern Italy, presented at age 16ys to the Ophthalmology Clinic, Policlinico Hospital, Italy, with a history of subacute, painless, and rapidly progressive bilateral vision loss. At the time of presentation, the proband appeared to be healthy, a well-developed boy but with clear signs of anxiety. One month prior to presentation, he had noticed impaired sight at his right eye and, within a few days, he could only see shadows. One week after the loss of sight in his right eye, the same symptom occurred in his left eye. Ophthalmologic examinations, at the moment of hospitalization, revealed at right eye (RE), BCVA of 20/200; hyperemic optic disk, tortuosity, and telangiectasia of retinal vasculature and absence of leakage and staining of the retinal vessels were revealed using fluorescein angiography. OCT examination showed an increase of nerve fibers layer thickness (average RNFL 108.58) and CVC examination revealed a deep central scotoma at RE, while there was not any alteration at left eye (LE). After six weeks, the young man presented the same symptomatology at his LE. Progressively, both optic nerves developed atrophy that was documented by OCT examination. CVC examination was no more possible because the visual acuity dropped to only light perception. All the family members underwent a complete ophthalmological examination. Visual acuity was 20/20 in all members. *Fundus* examination showed a hyperemic optic disk and vessels tortuosity in the proband’s young brother (III:2) and sister (III:3), whereas it was normal for the other family members. Visual field analysis was performed in all the available family members (Fig. 1) and resulted within normal ranges as well as the OCT-RNFL thickness examinations that were also in the normal range (NR: 97.3−/+ 9.6 μm). After disease onset, the proband started ubiquinone analog therapy with *Idebenone* (100 mg b.i.d.) without any improvement of visual acuity. Additionally, the proband had experienced epilepsy at 13 years of age, with recurrent tonic-clonic seizures that were under control with Oxcabazepine tablets 1800 mg/day, Topiramate tablets 400 mg/day, paroxetine hydrochloride tablets 20 mg/day, Pregabalin tablets 450 mg/day, Lorazepam tablets 1 mg/day as needed. He had no history of smoking, alcohol and of any illicit drug use. Interestingly, family history was significant for intellectual disability and hyperactivity in the mother (II:1) and siblings (III:2 and III:3) (Fig. 1) as outlined below; no one of the family used tobacco or alcohol with the exception of II:3 who referred tobacco abuse (Fig. 1).

To measure intelligence as IQ score in agreement with the age of patients, the Wechsler Intelligence Scalefor Children - Revised (WISC-R) was used for subjects aged 6 to 16 years, whereas the Wechsler Adult Intelligence Scale - Revised (WAIS-R) for subjects aged 17 to 90 years. The test showed that the proband manifested mild intellectual disability, whereas his available relatives all resulted in borderline intellectual functioning (Table 1). Recently, the youngest brother (III-2), who was suffering from severe migraine, at the age of 20ys, has developed recurrent seizures such as the proband, that are under control by Clonazepam 2.5 mg drops (12 drops/day) and Lacosamide tablets 400 mg/day. This study was conducted with the approval of the Institutional Review Board of Azienda Consorziale Policlinico Bari and Bari University. Before recruitment into our study, all participants and their guardians signed written informed consent in accordance with the guidelines of the Declaration of Helsinki. This research abided by the process of collection of data from patients with genetic diseases and the requirements of the Italian Ministry of Public Health. Written informed consent was obtained from the participant or from their parents for publication of this Case report.

**Table 1:** Clinical and genetic findings in LHON family

Subject ID	Sex	Age, y	Age at onset, y	Visual condition at the first examination		Current visual condition		Idebenone treatment	Recovery	Neuropsychological assessment				Neurologicalcomplication	m.3460A > G (% het)	m.961delTinsC_n_	Copy number (mtDNA/nDNA)
				RE	LE	RE	LE			Full scale scores	IQ profile						
											IQV	IQP	IQT				
*I-2*	F	71	70	20/20	20/20	20/20	20/20	–	–	–	–	–	–	–	HET (30%)	*n* = 4	206 ± 15
*II-1*	F	47	39	20/20	20/20	20/20	20/20	–	–	Mild intellectual disability (50–69)	59	68	59	N	HOM	*n* = 5	713 ± 144
*II-2*	F	46	38	20/20	20/20	20/20	20/20	–	–	Borderline intellectual functioning (70–84)	74	78	73	N	HET (40%)	n = 5	636 ± 74
*II-3*	M	44	43	20/20	20/20	20/20	20/20	–	–	–	–	–	–	N	HOM	*n* = 6	213 ± 9
*III-1*	M	25	17	20/200	20/200	20/800	Light perception	Y	N	Mild intellectual disability (50–69)	53	–	53	Epilepsy; Migraine	HOM	*n* = 9	240 ± 86
*III-2*	M	21	20	20/20	20/20	20/200	20/200	–	–	Borderline intellectual functioning (70–84)	66	82	72	Epilepsy Migraine	HOM	*n* = 7	604 ± 149
*III-3*	F	18	12	20/25	20/25	20/25	20/25	–	–	Borderline intellectual functioning (70–84)	84	86	84	N	HOM	*n* = 8	667 ± 72
*III-4*	M	18	10	20/20	20/20	20/20	20/20	–	–	Borderline intellectual functioning (70–84)	67	78	76	N	HET (40%)	n = 7	739 ± 184

Ophthalmological findings, extraocular signs, demographical data, genetic diagnosis and possible exposure to environmental risk factors and neuropsychological assessment are presented

The test is composed of verbal and performance scales divided into subtests in agreement with the different contents and type of intellectual operation required. The result is composed of three different scores: a total IQ (*IQT*), a Verbal IQ (*IQV*) and a Performance IQ (*IQP*). The two scales can be used separately to see if a person has particular strengths or weaknesses. The design of the test, with two scales, means that the verbal and performance scales can be independently used. Scale scores in the Verbal battery represent the sum and are converted to a *Verbal IQ score*; the same is done for the Performance scale scores that yield to the *Performance IQ score*. In turn, the *Verbal and Performance IQ scores* are overall converted to obtain the *Full-Scale IQ score*. The *Verbal*, *Performance* and *Full-Scale IQ scores* are normal IQs having a mean of 100 ± 15sd

*M* male, *F* female, *HOM* Homoplasmic, *HET* Heteroplasmic, *N* no, *Y* yes, *n.a.* not available, *RE* right eye, *LE* left eye, *IQT* total IQ, *IQV* verbal IQ, *IQP* performance IQ

Total genomic DNA was extracted by standard methods from peripheral blood of the patient and his relatives and from control subjects. Mitochondrial DNA genetic analysis was positive for the LHON m.3460G > A primary mutation in the proband and in all the family members; the mutation resulted as homoplasmic in the proband as well as in four LHON unaffected (II-1; II-3; III-2; III-3) while it was heteroplasmic in three LHON unaffected (I-2; II-2; III-4) subjects Table 1**;** showing a mean frequency of the mutant allele of 35% (*range 30–40%)* (Fig. 1).

We then measured mtDNA copy number, estimated as mtDNA/nDNA *ratio* [22], in peripheral blood samples from the proband and unaffected family members and then they were compared to control group’s (Table 1). The control group consisted of 90 unrelated subjects who had no history of a retinal disease, eye trauma or surgery, nor any evidence of systemic or neurological disease. MtDNA copy number of the homoplasmic relatives was evaluated in previous work [19]. Frequency distribution of the mtDNA copy number showed that the peak of mtDNA content shifted progressively towards higher values from control (210 ± 86) to affected (240 ± 86) to unaffected (548 ± 217) subjects with very high statistical significance: controls versus unaffected subjects, *P* < 0.001; proband versus unaffected subjects, *P* < 0.001 (ANOVA test). Furthermore, though the limitation of the sample size, when we compared mtDNA copy numbers between subjects harboring homo- or heteroplasmic m.3460G > A mutation, no difference was observed.

**Fig. 2:**
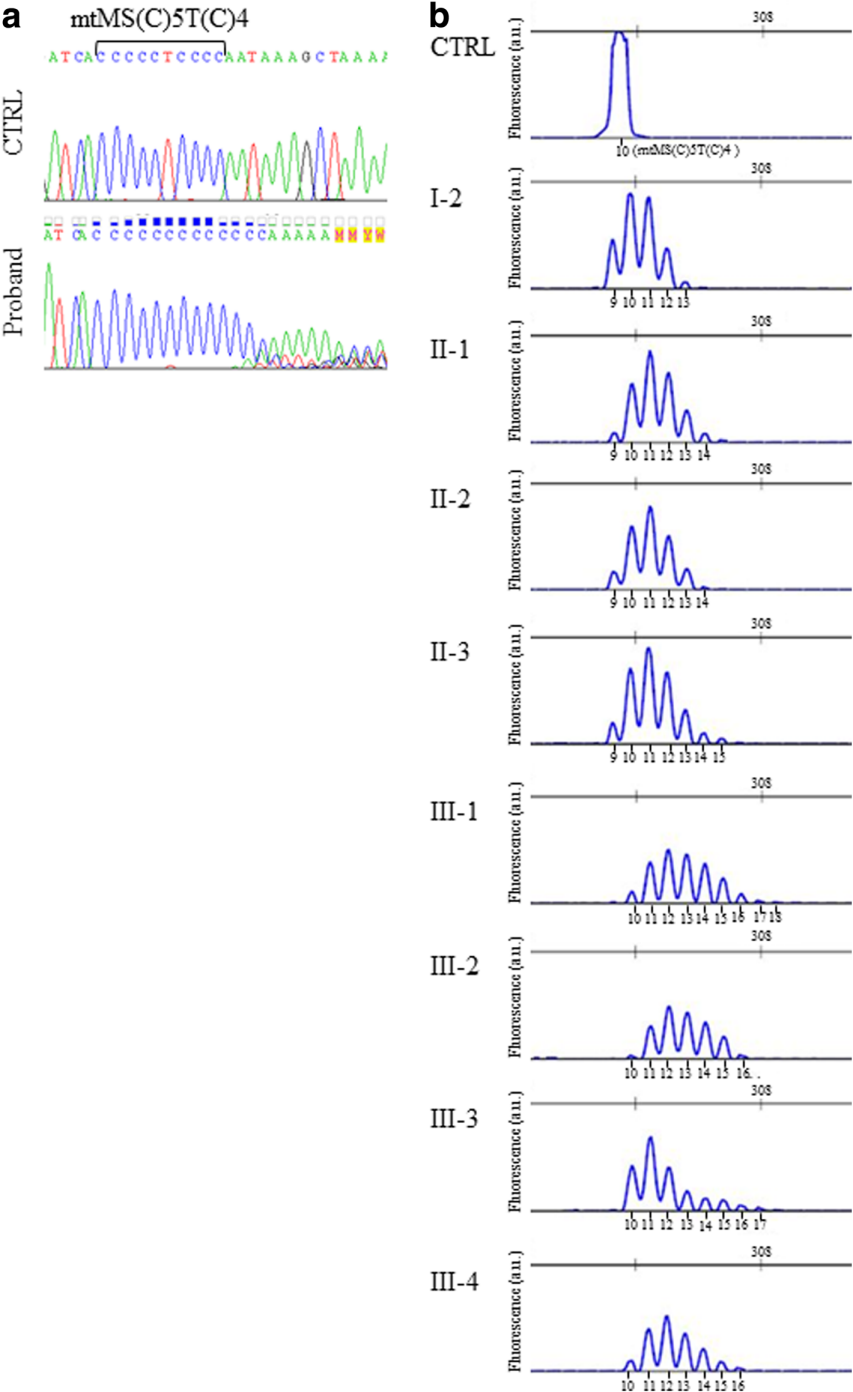
Analysis of the C-microsatellite in LHON family. **a** Sanger sequencing of the mtMS region of the proband and control**. b** Fragment analysis of mtMS in all the maternal family members

We considered the peculiarity of the clinical presentation of the proband, so we reasoned that additional mutations might contribute to the phenotype; we performed Sanger sequencing of the entire mtDNA genome [23]. All nucleotide variants were annotated according to the procedure described in *MtoolBox* [24] and are reported in Additional file 1. We identified 55 variants of which 46 contributed to defining the haplogroup U4a1a and 19 variants were prioritized (Additional file 1**)**. Interestingly, the proband showed multiple species of mtDNA molecules of variable lengths due to the variability of the number of cytosines inserted in the microsatellite at position m.961 of the *MTRNR1* gene which normally contains a (C)5 T(C)4 poly-cytidine tract (NC_012920.1; www.ncbi.nlm.nih.gov). We performed Sanger sequencing of *MTRNR1* region in all available relatives, confirming also in these, the microsatellite instability. The specific m.961delT + C(n)ins variant is annotated in *Mitomap* database (www.mitomap.org) mostly associated to deafness, but no population data are available for it and for variants harboring insertion with a number of C more than 7 (dbSNP link: https://www.ncbi.nlm.nih.gov/variation/view/?chr=MT&from=961&to=961&mk=961%3A961%7CNC_012920.1&assm=GCF_000001405.25). To discriminate among the different lengths of molecules carrying C(n)-microsatellite (mtMS) we cloned the proband appropriate DNA regions and then picked different clones for direct sequencing. We found eight additional species carrying the insertion of 1 to 8Cs starting at position m.961 of the wild-type molecule, which corresponds to a microsatellite of 10 to 17(C). To better investigate on the mtMS instability (mtMSI), fragment analysis was performed in all the available family members. This analysis showed that the *MTRNR1* mtMSs may have different lengths and that is heteroplasmic with variable percentages (Fig. 2; Additional file 2). I-2 had from 9 to 13(C); II-1, II-2, and II-3 had from 9 to 14(C) and 15(C), respectively; III-1 (proband), as well as III-2, III-3 and III-4 had from 9 to 16 (C); III-1 and III-3 showed also additional peaks i.e. 17(C) and 18(C). Quantitative analysis considering both the area and the height of the peaks showed that the 10 (C) microsatellite was the most represented species in I:2; in the second generation, there is a prevalence of 10–12(C) species, whereas in the third generation, there is a decrease of 10–12(C) species amount, with the exception of III-3, and a surge of 13(C) and especially of 14–16(C) and even of 17 and 18(C) which, on the contrary, were not at all present in the second generation (Additional file 2). In order to investigate the possible functional effect of the C-microsatellite expansion, the prediction of *MTRNR1* secondary structure and folding performed by *RNA fold* software from *Vienna RNA package* [25], assessed that m.961delT + C(n)ins would expand the size of a proposed loop structure in the *MTRNR1*.

## Discussion and conclusions

We identified an LHON pedigree characterized by intellectual disability, migraine, and epilepsy as extra-ocular signs which harbor a novel association of the m.3460G > A mutation with the instability of the C-microsatellite in the *MTRNR1* gene. In the LHON family, the m.3460G > A primary mutation was homoplasmic in the proband and either homo- or heteroplasmic in the seven LHON unaffected relatives. The heteroplasmic LHON unaffected subjects showed a mean percentage of 35% of mutated respect to total mtDNA, which is far below the previously reported 60% threshold predicted to represent the risk of visual loss [15]. Such evidence might be compatible with the notion true for the vast majority of mitochondrial diseases that the presence of wild-type in coexistence with mutated mtDNA might protect from the vision injury [15]. Nevertheless, the identification in four unaffected relatives (II-1; II-3; III-2; III-3) of homoplasmic LHON mutation annulled the hypothesis that the presence of wild-type together with m.3460G > A mutant alleles could be protective for visual loss. This suggests that additional protective factors must be considered. Furthermore, we found that the proportion of the m.3460G > A mutant allele does not increase in the successive generations (I-1: 30%; II-2: 40%; III-4: 40%) suggesting a random genetic drift mechanism behind the transmission of the heteroplasmic LHON mutation [26].

To the aim of investigating on further protective/risk factors for explaining the variability of LHON penetrance, we evaluated mtDNA content in peripheral blood cells in all the family members. We found a significant and consistent increase of mtDNA in unaffected subjects respect to the proband and controls which was independent of the homo/heteroplasmic *status* of the m.3460G > A mutation. Recently, comparison of mtDNA copy number from peripheral blood cells of unaffected, affected, and control subjects have shown that unaffected subjects have a significant increase of mitochondrial content per cell suggesting that it may be protective by a compensatory response to respiratory chain dysfunction [18–21]. Indeed, we found two exceptions to this general ‘rule’: the grandmother’s (I-2; 70ys) copy number was as low as the subject (II-3) and similar to normal controls’. Indeed, this result agrees with the knowledge that aging [22] as well as low estrogen condition, such that of a 70 yr-old woman [18, 19], are conditions known to determine a decrease in mtDNA content. The second case referred tobacco abuse; smoke has been shown to reduce mtDNA copy number in all cell types [27]. The evaluation of copy number in the peripheral blood was performed on samples obtained at first neurological exam of the family, before that the youngest brother (III-2) of the proband, developed decline of sight, migraine and seizures at the age of 20ys.

In the present family, LHON is accompanied by additional neurological and psychiatric manifestations, i.e. migraine, epilepsy and intellectual disability. Intellectual disability was present in all the maternal relatives. Formerly, in the original paper by Kwittken et al. 1958 some LHON patients were reported affected also by headache, vertigo, epilepsy, intellectual impairment, nystagmus, tremor, areflexia, loss of sphincter control, pyramidal tract disease, ataxia, or sensory disturbances among the LHON-associated extraocular signs [28]. Later, additional reports have provided evidence that LHON may manifest not exclusively as an ophthalmologic disorder but rather as a multisystemic disease with a predominant affection of the eyes [29, 30]. The genetic bases of the multisystem involvement had not been investigated thoroughly.

Intellectual disability, migraine, and epilepsy as extra-ocular signs were found in the herein described LHON pedigree of three generations. The proband and all the maternal relatives have, in addition to the m.3460G > A mutation, a heteroplasmic expansion of the poly-cytidine tract of the microsatellite at m.961 in *MTRNR1*gene. The m.961 C-stretch represents one of the overall 14 mtMSs found in the D-loop and in different coding regions of the mtDNA. This region is not evolutionarily strictly conserved among mammals but it is highly specific of *Homo sapiens* thus pointing to a functional restriction specific for humans [31]. Interestingly, it has never been associated with pathological conditions including intellectual disability. In the family, we found that the relative proportions of the C-microsatellite region lengths are maintained and expanded in maternally relatives suggesting that the first event of expansion had arisen in the grandmother - or possibly even before - and then had reached fixation within individuals as a consequence of misalignment and/or slippage during replication when stuttering can generate mixed population of C-mtMSs having different lengths. During the following generations, the transmission of the shortest mtMSs together with the expansion of mtMSs length may take place which generates heteroplasmy of the mtMS [32]. It has been proposed that m.961 position per se, either as transition or deletion in the middle of the C-microsatellite, may affect the tertiary or quaternary structure of the small ribosomal subunit thus impairing protein synthesis [33]. Moreover, once the G-rich region is transcribed in RNA, it may form a strong G-quadruplex structure leading to termination of RNA synthesis, as it occurs during primer formation for mtDNA replication [34]. If this mechanism takes place, we can hypothesize a reduction in rRNA steady-state level and ribosome assembly. But both hypotheses cannot be tested because we do not have cells deriving from the family members.

It is widely reported that *MTRNR1* gene is a hotspot for mutations associated with aminoglycoside ototoxicity [35–40]. The variable C-cluster of the *MTRNR1* region maps between 21 and 22 loops of the secondary structure of the ribosomal RNA [41] and has been implicated as having a role in the phenotypic expression of the m.1555A > G mutation in a large Chinese pedigree [42]. Deafness or hearing loss was not described among signs either in the proband or in his relatives but we cannot exclude that up to now no one had ever been exposed to aminoglycosides. Typically, intellectual disability may be part of a more complex syndromic condition and, to our knowledge, it has never been associated with mtDNA alterations. Though we cannot exclude that there might be a coincidence of an LHON mutation with a mutation in nuclear genes associated with intellectual disability [43] and epilepsy [44], we noticed that the neurologic and psychiatric signs manifested as a strong maternal inherited trait. The peculiar pattern of *MTRNR1* mutation as well as the presence of the longest species of mtMS segregating with intellectual disability is suggestive of their association although the size of the family does not allow to make a statistically significant link between the presence of the 961Tdel + C(n)ins variant and the non-LHON clinical features detected in the family. Therefore, the possible synergistic effect of the 961Tdel + C(n)ins with the 3460G > A remains an unproven hypothesis.

In conclusion, we report that the *MTRNR1* together with LHON primary mutation may complicate LHON phenotype sustaining the concept that the combinatorial effect of mitochondrial DNA mutations may have a synergistic role in worsening or widening the *spectrum* of LHON phenotype. In addition, we confirmed that increased mtDNA copy number is determinant in protecting from vision loss in presence of an LHON primary mutation.

## Additional files

Additional file 1: Prioritization of mtDNA variants by MToolBox in LHON proband. All potentially deleterious mutations not contributing to the macro-haplogroup definition and, if non-synonymous, predicted as disease-associated by at least one of the pathogenicity prediction methods are reported as prioritized. **Nt**, nucleotide; **AA**, amino acid; **dbSNP**, single nucleotide polymorphism database. (DOC 110 kb)

Additional file 2: Fragment analysis report. Report of height and area of the peak signals representing the PCR fragments analyzed (base pairs; bp); length fragment species are expressed as arbitrary unit. (DOC 205 kb)

## Abbreviations

BCVA: Best Corrected Visual Acuity
IQ: Intelligence Quotient
LE: Left eye
MTRNR1: Mitochondrial encoded 12S rRNA gene
OCT: Optical Coherent Tomography
RE: Right eye
RNFL: Retinal Nerve Fiber Layer Thickness
