# Supplementary material for: Leber’s hereditary optic neuropathy, intellectual disability and epilepsy presenting with variable penetrance associated to the m.3460G >A mutation and a heteroplasmic expansion of the microsatellite in MTRNR1 gene – case report
Source: BMC Med Genet. 2018 Jul 27;19:129. doi: 10.1186/s12881-018-0644-3 (PMC6062935; doi:10.1186/s12881-018-0644-3)
Supplement: Supplementary file 1 — Prioritization of mtDNA variants by MToolBox in LHON proband. All potentially deleterious mutations not contributing to the macro-haplogroup definition and, if non-synonymous, predicted as disease-associated by at least one of the pathogenicity prediction methods are reported as prioritized. Nt, nucleotide; AA, amino acid; dbSNP, single nucleotide polymorphism database. (DOC 110 kb) [file 12881_2018_644_MOESM1_ESM.doc]

**Additional file 1. Prioritization of mtDNA variants by MToolBox in LHON proband**

All potentially deleterious mutations not contributing to the macro-haplogroup definition and, if non-synonymous, predicted as disease-associated by at least one of the pathogenicity prediction methods are reported as prioritized. **Nt**, nucleotide; **AA**, amino acid; **dbSNP**, single nucleotide polymorphism database.

| **Haplogroup** | **Variant Allele** | ***Locus*** | **Nt Variability** | **Codon Position** | **AA Change** | **AA Variability** | **Disease Score** | **Mitomap Associated Disease(s)** | **Somatic Mutations** | **dbSNP ID** |
| --- | --- | --- | --- | --- | --- | --- | --- | --- | --- | --- |
| **U4a1** | 955.CCC | MT-RNR1 | 0.0 |  |  |  |  |  |  |  |
|  | 3460A | MT-ND1 | 0.00146 | 1 | A52T | 0.001 | 0.763 | LHON |  | rs199476118 |
|  | 1520C | MT-RNR1 | 0.00228 |  |  |  |  |  |  |  |
|  | 6975C | MT-CO1 | 0.00319 | 1 | syn | 0.0 |  |  |  |  |
|  | 12937G | MT-ND5 | 0.0134 | 1 | M201V | 0.0474 | 0.119 |  |  | rs201612920 |
|  | 16134T | MT-DLOOP | 0.0177 |  |  |  |  |  | Glioblastoma | rs369248643 |
|  | 8818T | MT-ATP6 | 0.0323 | 1 | syn | 0.0 |  |  |  |  |
|  | 6047G | MT-CO1 | 0.0544 | 3 | syn | 0.0034 |  |  | Pancreatic cancer cell line, glioblastoma |  |
|  | 11332T | MT-ND4 | 0.0553 | 3 | syn | 0.0 |  |  | Thyroid tumor | rs55714831 |
|  | 15693C | MT-CYB | 0.0556 | 2 | M316T | 0.0758 | 0.083 | Possibly LVNC cardiomyopathy-associated |  | rs200975632 |
|  | 14620T | MT-ND6 | 0.0577 | 3 | syn | 0.0 |  |  | Glioblastoma |  |
|  | 5999C | MT-CO1 | 0.0606 | 3 | syn | 0.0011 |  |  | Pancreatic cancer cell line, glioblastoma |  |
|  | 4646C | MT-ND2 | 0.061 | 3 | syn | 0.0016 |  |  | Glioblastoma |  |
|  | 513.CACA | MT-DLOOP | 0.0681 |  |  |  |  |  |  |  |
|  | 961C | MT-RNR1 | 0.0976 |  |  |  |  | DEAF, possibly LVNC-associated |  | rs3888511 |
|  | 16356C | MT-DLOOP | 0.121 |  |  |  |  |  | Glioblastoma | rs62581340 |
|  | 499A | MT-DLOOP | 0.129 |  |  |  |  |  | Thyroid & prostate tumors | rs3901846 |
|  | 310.C | MT-DLOOP | 0.283 |  |  |  |  |  |  |  |
|  | 1811G | MT-RNR2 | 0.326 |  |  |  |  |  | Head/neck tumor | rs28358576 |
