# Supplementary material for: Leber’s hereditary optic neuropathy, intellectual disability and epilepsy presenting with variable penetrance associated to the m.3460G >A mutation and a heteroplasmic expansion of the microsatellite in MTRNR1 gene – case report
Source: BMC Med Genet. 2018 Jul 27;19:129. doi: 10.1186/s12881-018-0644-3 (PMC6062935; doi:10.1186/s12881-018-0644-3)
Supplement: Supplementary file 2 — Fragment analysis report. Report of height and area of the peak signals representing the PCR fragments analyzed (base pairs; bp); length fragment species are expressed as arbitrary unit. (DOC 205 kb) [file 12881_2018_644_MOESM2_ESM.doc]

**Additional file 2. Fragment analysis report**

Report of height and area of the peak signals representing the PCR fragments detectable

(base pairs; bp); length of fragments are expressed as arbitrary units.

| ***Sample*** | ***Allele*** | ***Size (bp)*** | ***Height*** | ***Area*** |
| --- | --- | --- | --- | --- |
| ***I:1*** | *9C* | 299.72 | 3533 | 18962 |
|  | *10C* | 300.74 | 6863 | 46071 |
|  | *11C* | 301.79 | 6604 | 42060 |
|  | *12C* | 302.73 | 2901 | 15603 |
|  | *13C* | 303.78 | 577 | 1887 |
| ***II:1*** | *9C* | 299.81 | 748 | 2825 |
|  | *10C* | 300.85 | 4148 | 23913 |
|  | *11C* | 301.8 | 6507 | 41650 |
|  | *12C* | 302.85 | 4960 | 29455 |
|  | *13C* | 303.9 | 2234 | 11409 |
|  | *14C* | 304.85 | 724 | 2445 |
| ***II:2*** | *9C* | 299.82 | 1417 | 6908 |
|  | *10C* | 300.84 | 4561 | 28976 |
|  | *11C* | 301.79 | 6045 | 39787 |
|  | *12C* | 302.85 | 3950 | 24324 |
|  | *13C* | 303.9 | 1511 | 7504 |
|  | *14C* | 304.85 | 308 | 1021 |
| ***II:3*** | *9C* | 299.73 | 1487 | 6663 |
|  | *10C* | 300.73 | 5291 | 30835 |
|  | *11C* | 301.77 | 6697 | 44001 |
|  | *12C* | 302.82 | 5007 | 29296 |
|  | *13C* | 303.75 | 2366 | 11756 |
|  | *14C* | 304.79 | 836 | 3065 |

| ***Sample*** | ***Allele*** | ***Size (bp)*** | ***Height*** | ***Area*** |
| --- | --- | --- | --- | --- |
| ***III:1*** | *10C* | 300.73 | 744 | 2431 |
|  | *11C* | 301.78 | 2608 | 12690 |
|  | *12C* | 302.83 | 3377 | 18282 |
|  | *13C* | 303.77 | 3043 | 16215 |
|  | *14C* | 304.81 | 2518 | 11949 |
|  | *15C* | 305.85 | 1597 | 6663 |
|  | *16C* | 306.89 | 565 | 2042 |
| ***III:2*** | *10C* | 300.84 | 328 | 1021 |
|  | *11C* | 301.89 | 2186 | 11118 |
|  | *12C* | 302.93 | 3447 | 20856 |
|  | *13C* | 303.87 | 3053 | 17813 |
|  | *14C* | 304.91 | 2433 | 12870 |
|  | *15C* | 305.96 | 1499 | 6838 |
|  | *16C* | 306.89 | 449 | 1572 |
| ***III:3*** | *10C* | 300.84 | 3201 | 17165 |
|  | *11C* | 301.89 | 5215 | 33915 |
|  | *12C* | 302.83 | 3063 | 17561 |
|  | *13C* | 303.87 | 1413 | 6447 |
|  | *14C* | 304.91 | 984 | 4096 |
|  | *15C* | 305.96 | 854 | 3442 |
|  | *16C* | 306.89 | 506 | 2008 |
| ***III:4*** | *10C* | 300.73 | 934 | 3495 |
|  | *11C* | 301.77 | 3317 | 17281 |
|  | *12C* | 302.81 | 4280 | 23889 |
|  | *13C* | 303.74 | 2913 | 15235 |
|  | *14C* | 304.78 | 1774 | 7637 |
|  | *15C* | 305.82 | 857 | 3042 |
|  | *16C* | 306.85 | 411 | 1504 |
